# Etiologic subtype predicts outcome in mild stroke: prospective data from a hospital stroke registry

**DOI:** 10.1186/1471-2377-13-154

**Published:** 2013-10-24

**Authors:** Zilong Hao, Ming Liu, Deren Wang, Bo Wu, Wendan Tao, Xueli Chang

**Affiliations:** 1 From the Stroke Clinical Research Unit, Department of Neurology, West China Hospital, Sichuan University, No 37, Guo Xue Xiang, Chengdu 610041, China; 2 From the Stroke Clinical Research Unit, Department of Neurology, State Key Laboratory of Biotherapy and Cancer Center, West China Hospital, Sichuan University, No 37, Guo Xue Xiang, Chengdu 610041, China

**Keywords:** Acute ischemic stroke, Minor stroke, Etiology, Death/disability

## Abstract

**Background:**

Few studies on whether etiologic subtype can predict outcome in mild stroke are available. The study aim to explore the effect of different etiologic subtype on prognosis of these patients.

**Methods:**

We prospectively registered consecutive cases of acute ischemic stroke from September. 01, 2009 to August. 31, 2011. Patients with National Institute of Health Stroke Scale (NIHSS) ≦3 and within 30 days of symptom onset were included. All cause death or disability (defined as modified Rankin Scale >2) were followed up at 3 months. The multivariate logistical regression model was used to analyse relationship between etiologic subtype and clinical outcomes.

**Results:**

We included 680 cases, which accounted for 41.1% (680/1655) of the total registered cases. Mean age were 62.54 ± 13.51 years, and males were 65.4%. The median time of symptoms onset to admission was 72 hours. 3.8% (26/680) of cases admitted within 3 hours and 4.7% (32/680) admitted within 4.5 hours. However, no patient received intravenous thrombolysis. Of included patients, 21.5% large-artery atherosclerosis, 40.6% small-vessel disease, 7.5% cardioembolisms, 2.2% other causes and 28.2% undetermined causes. The rate of case fatality and death/disability was 2.2% and 10.1% respectively at 3 months. After adjustment of potential confounders, such as age, sex, NIHSS on admission and vascular risk factors et al., cardioembolism (RR = 3.395;95%CI 1.257 ~ 9.170) was the predictor of death or disability at 3 months and small vessel occlusion (RR = 0.412;95%CI 0.202 ~ 0.842) was the protective factor of death or disability at 3 months.

**Conclusion:**

Different etiologic subtype can predict the outcome in patients with mild stroke and it can help to stratify these patients for individual decision-making.

## Background

It is very common that acute ischemic stroke patients have mild symptoms or rapidly improving stroke symptoms, which makes the decisions of treatment in dilemma. Intravenous thrombolysis with recombinant tissue plasminogen activator (IV rtPA) is proven to be the most effective treatment for acute ischemic stroke [1,2]. However, only 1%-5% patients are treated with IV rtPA. In addition to the narrow time window for treatment, not treat patients with mild or rapidly improving symptoms is one important reason [3]. In studies evaluating eligibility for thrombolysis, up to 43% of patients with mild or improving stroke symptoms do not receive thrombolytic therapy because of clinicians think the natural course of these patients is benign [4]. However, according to recent reports, 15%–31% of patients with mild or rapidly improving symptoms have dependent or dead during hospital admission without thrombolysis [3,5-7]. Therefore, early identification of these patients with poor outcome will contribute to the choice of early intervention to prevent the occurrence of poor outcomes. Studies suggest that mild stroke patients with large vessel occlusion are at a high risk for early neurological deterioration or poor outcome [8]. Imaging with advanced MRI is one possibility to guide treatment decision in mild stroke [9-11]. Therapies directed at the underlying mechanism will be more effective. But there is still lack of clinical outcomes in patients with different etiologic subtype. This study aims to observe clinical characteristics and explore the effect of different etiologic subtype on prognosis of mild stroke.

## Methods

### Subjects

Due to this is an observational study that will only assess outcomes in participants, all participants or their designated relatives provided verbal informed consent for obtaining relative prognostic information. This research project was approved by the Scientific Research Department of West China Hospital, which conformed to the local ethic criteria for human researches. We included acute ischemic stroke admitted consecutively to neurological wards of the West China Hospital, Sichuan University within 30 days of symptom onset between September. 01, 2009 to August. 31, 2011. All patients had a clinical diagnosis of stroke according to World Health Organization (WHO) criteria and intracranial hemorrhage were further excluded by CT or MRI scan in the hospital [12]. Patients with National Institute of Health Stroke Scale (NIHSS)≦3 were included [13] in this study.

### Data collection

Details of patient demographies, vascular risk factors, Blood pressure (BP) on admission, laboratory test, brain image data and complications were recorded at the time of assessment using a standardized structured form. Vascular risk factors included hypertension (HTN), diabetes, dyslipidemia, coronary heart disease (CHD), history of stroke, status of smoking and alcohol. Severity of stroke was assessed using the NIHSS score. Medical complications include respiratory infection, urinary tract infection, electrolyte disturbances, hemorrhage of digestive tract and epilepsy. Determination of ischemic stroke etiology was according to the Trial of Org 10172 in Acute Stroke Treatment (TOAST) criteria: large-artery atherosclerosis (LAA); small artery occlusion (SAO); cardioembolism (CE); stroke of other determined cause (OC); and stroke of undetermined cause (UND) [14].

### Assessment of outcome

The main outcomes were death and disability at 3 month after onset. Death was all-cause case fatality. Disability was defined as modified Rankin Scale (mRS) > 2 [15]. Patients were followed-up by telephone call, clinic interview or letter inquiry. To ensure the quality of information collection, all investigators received training in the beginning according to our standard operational manual. All data were recorded in a structured data sheet. The outcome assessors were blinded to the information of patients. The poor outcome defined as death or disability (mRS >2) at 3 months.

### Statistical analysis

Differences between two groups were tested using t test, Mann–Whitney U test or Chi-Square test where appropriate. Variables that were identified as significant in the univariate analyses (P <0.10) were entered into multivariate logistic regression analyses. The multivariate logistical regression was used to analyze association between different etiologic subtype and clinical outcomes, adjusting for all potential confounders including age, sex, NIHSS on admission, vascular risk factors and complications et al. Data are reported with Relative Risk (RR) and 95% confidence intervals (CI). All statistical analyses were performed with SPSS for Windows, Version 16.0 (SPSS Inc.).

## Results

### Baseline characteristics

We included 1655 patients with acute ischemic stroke within 30 days of symptom onset. Of them, patients with mild stroke accounted for 41.1% (680/1655). The mean age was 62.54 ± 13.51 years (18–97 years). Males accounted for 65.4% (445/680). The median time of symptoms onset to admission was 72 hours (2–270 hours). The median of NIHSS on admission is 2(1–3). Of these patients, 21.5% large-artery atherosclerosis, 40.6% small-vessel disease, 7.5% cardioembolisms, 2.2% other causes and 28.2% undetermined causes. 3.8% (26/680) of cases admitted within 3 hours and 4.7% (32/680) admitted within 4.5 hours. However, no patient received rt-PA treatment. The common complications were 7.7% respiratory tract infection, 2.0% urinary tract infection, 2.2% electrolyte imbalances, 0.9% gastrointestinal bleeding and 0.4% secondary epilepsy. The proportion of case fatality and death/disability was 2.2% and 10.1% respectively at 3 months. In order to analyze the effect of specific etiology on prognosis of minor stroke, such as cardioembolisms, large-artery atherosclerosis, small-vessel disease, the patients with undetermined etiology were excluded from the following analysis. In addition, other causes were also excluded due to few patients were included in our study. So we keep a total of 453 cases for further analysis. Compared to patients without death or disability, patients with poor outcome were younger, had higher proportions of large-artery atherosclerosis or cardioembolisms and had high rate of complications of respiratory and urinary tract infection. Baseline characteristics of this cohort were described in Table 1.

**Table 1:** Characteristics of patients with minor stroke according to outcomes

**Variates**	**Death/disability N1 = 43**	**No death/disability N2 = 373**	**P value**
Age, years	68.84 ± 10.03	63.34 ± 12.61	0.006
Male, %	25(58.1)	257(68.9)	0.153
Time from onset, h	48	48	0.418
SBP on admission, mmHg	141.88 ± 25.96	143.40 ± 21.38	0.667
DBP on admission, mmHg	83.28 ± 16.99	84.13 ± 13.64	0.708
Hypertension, %	26(60.5)	222(59.5)	0.905
Diabetes mellitus, %	11(25.6)	70(18.8)	0.285
Dyslipidemia, %	3(7.0)	31(8.3)	1.000
Coronary heart diseas, %	2(4.7)	22(5.9)	1.000
History of ischemic stroke, %	1(2.3)	22(5.9)	0.493
History of hemorrhagic stroke	2(4.7)	3(0.8)	0.086
Alcohol, %	6(14.0)	80(20.3)	0.251
Smoking, %	13(30.2)	125(33.)	0.665
TOAST classification			0.055
LAA	16(37.2)	110(29.5)	
SAO	19(44.2)	228(61.1)	
CE	8(18.6)	35(9.4)	
Respiratory infection, %	10(23.3)	20(5.4)	0.000
Urinary tract infection, %	3(7.0)	5(1.3)	0.040
Electrolyte disturbance, %	0(0.0)	8(2.1)	1.000
Hemorrhage of digestive tract,%	1(2.3)	3(0.8)	0.355
Epilepsy, %	1(2.3)	1(0.3)	0.196

LAA: large-artery atherosclerosis.

SAO: small artery occlusion.

CE: cardioembolism.

### The influence of etiologic subtype on outcomes at 3 month

After adjustment of potential confounders, such as age, sex, NIHSS on admission and vascular risk factors et al., cardioembolism was the independent predictor of death or disability at 3rdmonth (RR = 3.395;95%CI 1.257 ~ 9.170) (Table 2). Small vessel occlusion also was the independent predictor of good outcome at 3rd month (RR = 0.412;95%CI 0.202 ~ 0.842) (Table 3). Large-artery atherosclerosis was not the independent predictor of death or disability, but had a trend of increasing death/disability (RR = 1.497; 95%CI 0.723 ~ 3.102) (data not shown). In addition, age, history of intracerebral hemorrhage and complications were independent predictive factor for death or disability at 3rd month across different etiologic subtype analysis.

**Table 2:** Multivariate logistic analysis of the influence of cardioembolism on death/disability

**Variates**	**RR**	**95% CI**	**P value**
Age	1.037	1.003 ~ 1.073	.031
Gender	.628	.276 ~ 1.426	.266
Time from onset	1.002	1.000 ~ 1.004	.107
SBP on admission	.992	.969 ~ 1.015	.486
DBP on admission	1.008	.972 ~ 1.045	.668
Hypertension	1.313	.610 ~ 2.826	.486
Diabetes mellitus	1.263	.533 ~ 2.989	.596
Hypercholesterolemia	1.188	.317 ~ 4.448	.798
Coronary heart diseas	.321	.060 ~ 1.708	.183
History of ischemic stroke	.228	.025 ~ 2.070	.189
History of hemorrhagic stroke	11.297	1.469 ~ 86.889	.020
Alcohol	.619	.208 ~ 1.848	.390
Smoking	1.620	.644 ~ 4.072	.305
CE VS. Non-CE	3.395	1.257 ~ 9.170	.016
Complications	3.405	1.527 ~ 7.596	.003

CE: cardioembolism.

**Table 3:** Multivariate logistic analysis of the influence of SAO on death/disability

**Variates**	**RR**	**95% CI**	**P value**
Age	1.035	1.002 ~ 1.069	.039
Gender	.614	.270 ~ 1.398	.245
Time from onset	1.001	.999 ~ 1.003	.306
SBP on admission	.987	.965 ~ 1.010	.256
DBP on admission	1.014	.978 ~ 1.051	.449
Hypertension	1.428	.659 ~ 3.095	.367
Diabetes mellitus	1.276	.536 ~ 3.041	.582
Hypercholesterolemia	1.114	.301 ~ 4.124	.871
Coronary heart diseas	.437	.088 ~ 2.170	.311
History of ischemic stroke	.265	.029 ~ 2.425	.240
History of hemorrhagic stroke	12.420	1.423 ~ 108.367	.023
Alcohol	.628	.209 ~ 1.886	.407
Smoking	1.600	.630 ~ 4.063	.323
SAO VS. Non-SAO	.412	.202 ~ .842	.015
Complications	3.477	1.536 ~ 7.869	.003

SAO: small artery occlusion.

## Discussion

In this study, we found patients with mild stroke accounted for 41.1% in our cohort. The proportion of case fatality and death/disability was 2.2% and 10.1% respectively at 3 month. Different etiologic subtype can predict the outcome in these patients.

Previous studies showed the proportion of mild stroke veried from 5.8% to 62.3% based on different criteria, which in accordance with our study [16,17]. Khatri et al. suggests that approximately one third of so-called mild strokes have significant disability (mRS 2–6) that persists at 3 months [7]. In contrast, we found 10.1% of minor stroke had death or disability. The reasons of low proportion of our study may be as follows: (1) they used the NIHSS≦5 as criteria whereas we used NIHSS≦3, which indicates they included participants more severer than ours; (2) they measured disability with mRS > 1 whereas we defined it as mRS > 2; (3) they enrolled patients within 24 hours whereas we included them within 30 days, which means their participants may be prone to have early worsening or early recurrence than ours; (4) In general, the death or disability of acute ischemic stroke patients in China is lower than abroad [18,19], so may be minor stroke no exception.

Several studies reported the predictors of poor outcome of mild stroke, such as age, diabetes, coronary heart disease, smoking, and vessel stenosis or occlusion were predictor of death or disability or recurrence at the end of follow up period [20-25]. Ferrari J et al. found hypertension, diabetes, cardiac decompensation, acute infection, and stroke etiology (LAA and CE) emerged as independent risk predictors for early deterioration in patients with a transient ischaemic attack (TIA) or minor ischemic stroke, defined by an NIHSS score <4 [26]. In our cohort, age, history of intracerebral hemorrhage, infections and etiologic subtype (CE and SAO) were the independent predictors of death or disability at 3rd month. Our finding of etiologic subtype can predict outcome in mild stroke is more compelling, because consecutive subjects that were assessed beyond hospital discharge at 90 days. So far, although there are no identical variates for predicting the poor outcome of patients with minor stroke, future studies are needed to focus on how to really identify minor stroke patients with poor outcome by clinical features combined with imaging features. Recently, Strbian D et al. indicates that half of patients presenting with NIHSS 0–6 developed an infarction despite thrombolysis, and 40% had poor outcome. Perhaps, urgent multimodal imaging can help to identify mild stroke at risk of worsening [27]. As we known, etiologic subtype help stratify patients for secondary prevention and it may also be used for identifing poor outcome in patients with mild stroke.

At present, whether patients presenting with mild stroke should or should not be treated with intravenous rtPA is still unclear. In our study, 3.8% (26/680) of cases admitted within 3 hours and 4.7% (32/680) admitted within 4.5 hours. However, no patient received intravenous thrombolysis. In the light of the natural history of course of mild stroke is not always benign, before a large randomized trial proving its lack of benefit in mild stroke patients, IV t-PA should be considered in these patients.

Our study has several limitations. (1) It was performed in a single-center, which may not represent for whole Chinese population; (2) there is no consensus definition of mild stroke. The National Institute of Neurological Disorders and Stroke (NINDS) rtPA study and the European Cooperative Acute Stroke Study (ECASS) III both excluded patients with mild stroke, but they failed to clearly define a certain threshold for a mild stroke. We used diagnostic criteria NIHSS score ≦3, which be evaluated as the more suitable cutoff point [13]; (3) The effects of potential unknown confounders cannot be ruled out in our cohort. Despite these limitations, our study explored the relationship between etiologic subtype and outcomes by inclusion of propectively consecutive patients and the blind evaluation of the study outcome.

## Conclusions

In conclusion, our study indicates that different etiologic subtype can predict the outcome in patients with minor stroke it can help to stratify these patients for individual decision-making.

## Abbreviations

IV rtPA: Intravenous thrombolysis with recombinant tissue plasminogen activator; WHO: World Health Organization; NIHSS: National Institute of Health Stroke Scale; BP: Blood pressure; HTN: Hypertension; CHD: Coronary heart disease; TOAST: Trial of Org 10172 in Acute Stroke Treatment; LAA: Large-artery atherosclerosis; SAO: Small artery occlusion; CE: Cardioembolism; OC: Stroke of other determined cause; UND: Stroke of undetermined cause; mRS: modified rankin scale; TIA: Transient ischaemic attack; NINDS: National Institute of Neurological Disorders and Stroke; ECASS: European Cooperative Acute Stroke Study.

## Competing interests

The authors declare that they have no competing interests.

## Authors’ contributions

ZH was responsible for the conception and design of the study, data collection, data analysis and interpretation, drafting and revising the manuscript. ML was responsible for the conception and design of the study, data analysis and interpretation, and revising the manuscript. DW, BW, WT and XC were responsible for data collection, revising the manuscript. All authors have read and approved the final manuscript.

## Pre-publication history

The pre-publication history for this paper can be accessed here:

http://www.biomedcentral.com/1471-2377/13/154/prepub
